# Next‐Generation Dual 1550‐nm Erbium Glass Fiber and 1927‐nm Thulium Fiber Non‐Ablative Fractional Laser System: Provider and Patient Perspectives

**DOI:** 10.1111/jocd.71115

**Published:** 2026-09-02

**Authors:** Ashish C. Bhatia, Jeffrey S. Dover, Paul M. Friedman, Anne Chapas, Jill Waibel, Mitchel P. Goldman, Suzanne L. Kilmer, Su Yong Choi, Abby Jacobson

**Affiliations:** ^1^ Oak Dermatology Naperville Illinois USA; ^2^ SkinCare Physicians Chestnut Hill Massachusetts USA; ^3^ Dermatology & Laser Surgery Center Houston Texas USA; ^4^ UnionDerm Union Square Laser Dermatology New York New York USA; ^5^ Miami Dermatology & Laser Institute Miami Florida USA; ^6^ Cosmetic Laser Dermatology: A Platinum Dermatology Partners Company San Diego California USA; ^7^ Laser & Skin Surgery Center of Northern California Sacramento California USA; ^8^ Bausch Health Companies Inc Bridgewater New Jersey USA

To the Editor,

The next‐generation dual 1550‐nm erbium glass fiber and 1927‐nm thulium fiber non‐ablative fractional laser system (Fraxel FTX system; Solta Medical, Bothell, WA) was launched in 2025. Compared with the previous‐generation Fraxel DUAL system, enhancements include a lighter and more ergonomic handpiece, integrated tubing with directed cooling, a redesigned treatment tip, an enhanced user interface, and an updated optical tracking system. The system operates at the same wavelengths that, in a previous study, were associated with a quartile clinical improvement score of 1.9 (1 = 1%–25% improvement; 2 = 26%–50% improvement) for photodamage and pigmentation after ≤ 4 sessions [1]. The 1550‐nm laser coagulates the epidermis and dermis with ≤ 1.6‐mm depth of penetration, addressing severe sun damage, wrinkles, acne scars, and surgical scars. The 1927‐nm laser coagulates the epidermis and superficial dermis with ≤ 0.3‐mm depth of penetration, addressing mild‐to‐moderate sun damage, pigmented lesions, skin texture, and actinic keratosis [2].

A beta‐testing survey was conducted among 22 providers who treated 97 patients with the next‐generation device. Reflecting real‐world clinical practice, treatment protocols and posttreatment regimens were not standardized, enabling providers to customize treatments according to their own clinical experience and judgment, patient tolerability, skin condition, Fitzpatrick skin type, and body area. Providers assessed efficacy via Investigator Global Aesthetic Improvement Scale (I‐GAIS; 1 = worse; 5 = significant improvement) and quartile improvement scores (0 = no improvement; 4 = 76%–100% improvement), monitored safety, and answered questions about user experience. Patients completed surveys about comfort and satisfaction immediately after treatment (*n* = 85), during the healing process (7–14 days after treatment; *n* = 65), and 3–8 weeks after treatment (*n* = 38) and rated esthetic improvement via Patient Global Aesthetic Improvement Scale (P‐GAIS). Outcomes were analyzed on an as‐observed basis.

Patients were predominantly female (89%) and White (73%), with most having a Fitzpatrick skin type of II (53%). Common treatment objectives were dyschromia and fine lines and wrinkles. Patients were treated with the 1550‐nm laser (14%), 1927‐nm laser (40%), or both (45%).

The average I‐GAIS score after treatment was 3.4 (slight‐to‐moderate improvement), and the average quartile improvement score was 2.1 (26%–50% improvement; Figure 1A). No unexpected skin reactions or serious adverse events were reported. Most providers were “satisfied” or “very satisfied” with the system (91%) and noted it was “easy” or “very easy” to use (95%). Most providers (73%–96%) reported improvements in cable bundling, ergonomics, and tip design (Figure 1A). Additionally, 50% of providers noted that the new tracking system reduced treatment time by up to 20%.

**FIGURE 1:**
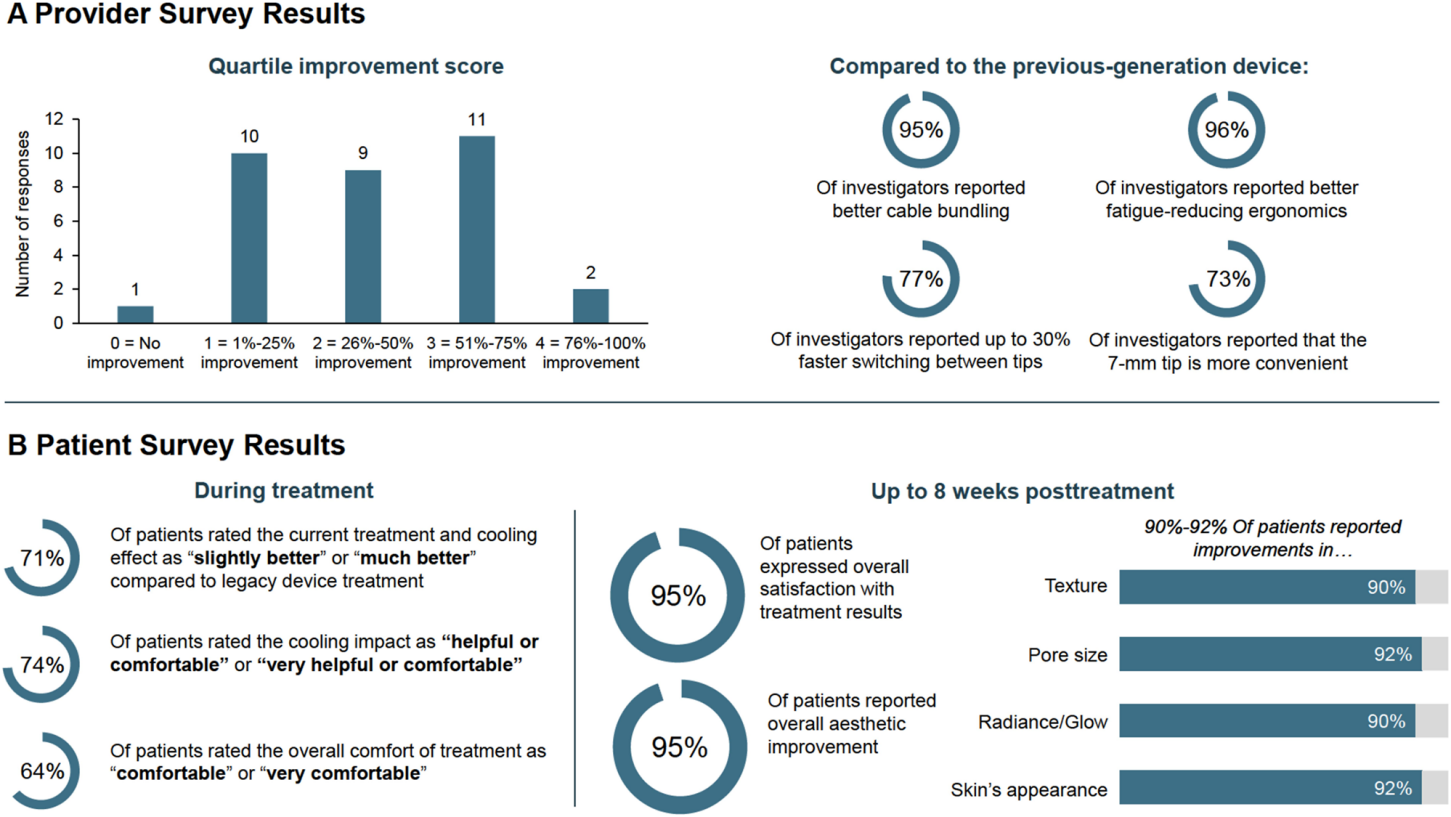
Responses to survey questions regarding (A) provider and (B) patient experiences with the next‐generation dual 1550‐nm erbium glass fiber and 1927‐nm thulium fiber non‐ablative fractional laser system.

Most patients (74%) rated the cooling effect as helpful in improving comfort during treatment, with 64% reporting their treatment as comfortable (Figure 1B). Patients with prior treatment with the legacy device (*n* = 42) generally reported a better treatment experience with the next‐generation device (slightly better, 21%; much better, 50%). Most patients (62%) noted the healing process was “comfortable” or “very comfortable,” with no‐to‐minimal pain (88%) and discomfort (75%). Additionally, 91% were “satisfied” or “very satisfied” with the overall treatment experience. In the weeks after treatment, 95% of patients reported overall esthetic improvement and overall satisfaction with treatment results, including improvements in skin texture, pore size, radiance/glow, and skin appearance. Lastly, 87% of patients expressed interest in receiving annual treatment. Patient‐reported maximum severity ratings for redness, itching, and peeling collected 7–14 days after treatment were moderate (72%), none to minimal (63%), and moderate (51%), respectively. Representative patient photographs are shown in Figure 2. These patients indicated the results were worth the cost and healing process.

**FIGURE 2:**
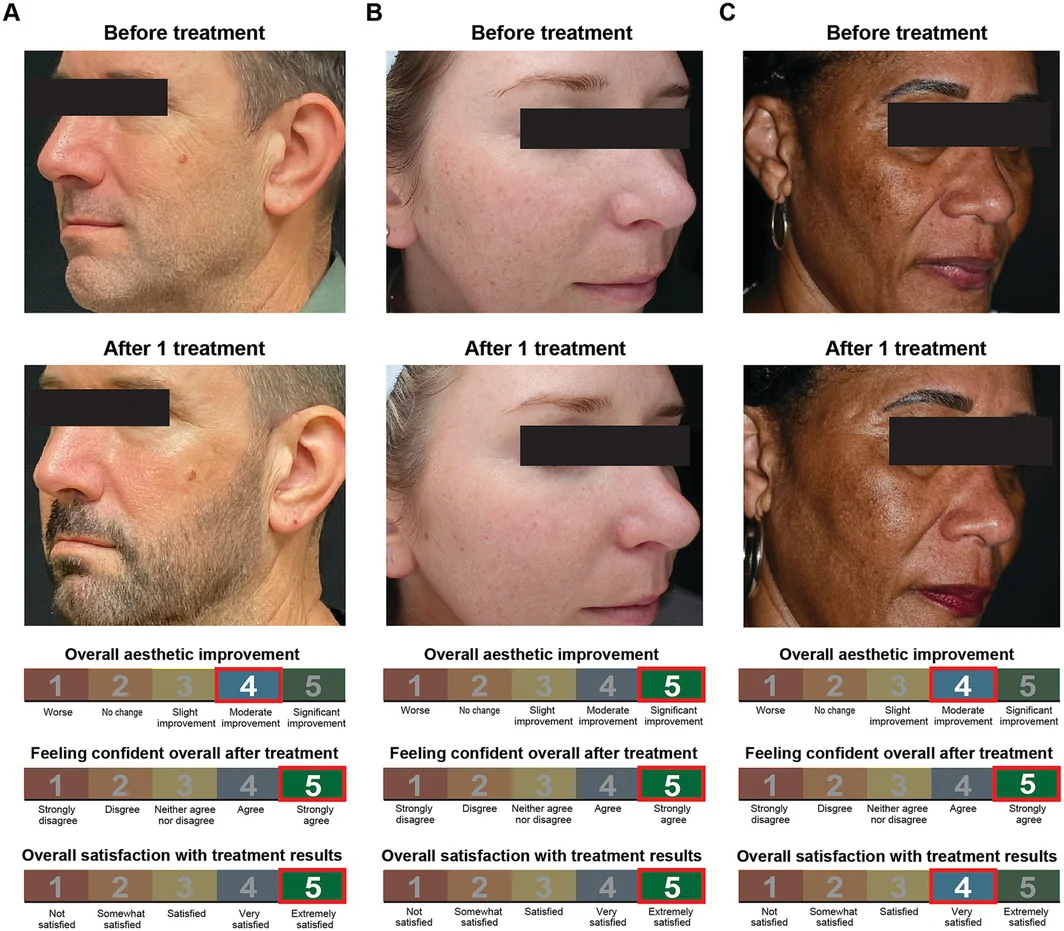
Representative patient photographs showing esthetic improvement with the next‐generation dual 1550‐nm erbium glass fiber and 1927‐nm thulium fiber non‐ablative fractional laser system, including improvement in (A) rhytids in a patient with FST II, (B) dyschromia and fine lines in a patient with FST II, and (C) melasma in a patient with FST V. “After” photos were taken 4 to 6 weeks after treatment. Patient images provided with written consent and courtesy of Dr. Suzanne L. Kilmer (panel A), Dr. Anne Chapas (panel B), and Dr. Jill Waibel (panel C). FST, Fitzpatrick skin type.

Overall, positive responses were reported for the next‐generation device, including enhanced ergonomics, features that reduced treatment time, high patient‐reported comfort and satisfaction, and esthetic improvement. Limitations included few standardized enrollment criteria that resulted in a population with minimal esthetic concerns and low potential for improvement and possible response bias related to responder attrition, although provider‐assessed outcomes did not differ by patient survey response status. This analysis suggests that the next‐generation 1550/1927‐nm non‐ablative fractional laser system represents an updated technological iteration of an existing, well‐established tool with a demonstrated safety profile.

## Author Contributions

A.C.B., J.S.D., P.M.F., A.C., J.W., M.P.G., and S.L.K. contributed to the investigation, writing – review and editing, and resources (provision of patients). S.Y.C. contributed to the formal analysis, data curation, and visualization. A.J. contributed to conceptualization, methodology, resources (study materials and instrumentation), writing – review and editing, supervision, project administration, and funding acquisition. All authors approved the final submitted version of the manuscript for publication.

## Funding

This study was sponsored by Solta Medical Inc., an affiliated company of Bausch Health Companies Inc.

## Ethics Statement

The authors have nothing to report.

## Consent

Written consent was given for the publication of anonymized patient images.

## Conflicts of Interest

A.C.B. has received grants or contracts, consulting fees, or honoraria from Arcutis, Avita Medical, Bausch Health, Bristol Myers Squibb, Cutera, Cytrellis, Elsevier, GLO Pharma, Hexagon Health Solutions, Lutronic/Cynosure, Masters of Aesthetics, MiMedx Group, Mooreland Associations, Northwestern Medical Faculty Foundation, Pfizer, RBC Consultants, Regeneron, Revance, Scientific Global Communications, Solta Medical, Strata Skin Sciences, and Theravant; has served as a board member or scientific board member for American Society for Dermatologic Surgery, American Society for Laser Medicine and Surgery, Bausch Health, Cutera, and Theravant; and may hold stock in Bausch Health, Cytrellis, GLO Pharma, Solta Medical, and Theravant. J.S.D. has received grants or research support from Allergan/AbbVie, Bausch & Lomb, Cutera, Cynosure, Evolus, InMode, and Revance; has been granted use of equipment from Allergan/AbbVie, Cutera, and Cynosure; has equity in Controversies & Conversations; and has served as a consultant to Allergan/AbbVie, Bausch & Lomb, Cutera, Cynosure, Fount Bio, Revance, UpToDate, and Vyome. P.M.F. has served on advisory boards for Acclaro Corp, Allergan, Candela Medical, Cytrellis, and Solta Medical; has received grants from Acclaro Corp and Bausch Health; and has received honoraria from Candela Medical and Solta Medical. A.C. has received honoraria or research funding or served on advisory boards for Allergan, Cytrellis, Evolus, InMode, Lumenis, Lutronic, Revance, Sofwave, Solta Medical, and Syneron Candela. J.W. has received research grants or consulting fees or served on scientific advisory boards for Allergan, Amgen, ArgenX, Bellanna, Bristol Myers Squibb, Candela, Cytrellis, Eli Lilly, Galderma, Janssen J&J, Lumenis, Neuronetics, Pfizer, P&G, RegenX, Sanofi, Shanghai Biopharma PWB, SkinCeuticals, and Solta Medical. M.P.G. has no disclosures relevant to this work to declare. S.L.K. has served on advisory boards for Acclaro Corp, Allergan, Cytrellis, Galderma, Lumenis, Revance, Sofwave, and Solta Medical. S.Y.C. and A.J. are employees of Bausch Health Companies Inc.

## Data Availability

The data that support the findings of this study are available from the corresponding author upon reasonable request.
